# Interobserver agreement: Individual CTG features show better agreement among investigators than the overall CTG assessment in cases of meconium-stained amniotic fluid

**DOI:** 10.18332/ejm/215682

**Published:** 2025-12-31

**Authors:** Linas Rovas, Meile Minkauskiene, Kristina Berskiene, Vaiva Maciulionyte, Akvile Papievyte, Ruta Petkeviciute, Augusta Petrusaite, Agne Pinauskaite

**Affiliations:** 1 Klaipeda University Hospital, Klaipeda, Lithuania; 2 Klaipeda State College, Klaipeda, Lithuania; 3 Lithuanian University of Health Sciences, Kaunas, Lithuania; 4 Hospital of Lithuanian University of Health Sciences Kauno Klinikos, Kaunas, Lithuania

**Keywords:** cardiotocography, reliability, interobserver agreement, fetal heart rate, intrapartum fetal monitoring

## Abstract

**INTRODUCTION:**

The objective of this investigation was to evaluate the interobserver agreement between different investigators on selected cardiotocogram (CTG) parameters.

**METHODS:**

Medical records were selected from birth histories of cephalic deliveries with meconium-stained amniotic fluid. A total of 84 CTGs were recorded and analyzed by six clinicians. Agreement metrics such as proportion of agreement (Pa) with corresponding 95% confidence intervals (95% CIs) and reliability indices calculated via the Fleiss kappa statistic, were employed to quantify interobserver consistency.

**RESULTS:**

CTG parameters baseline rate, variability, presence or absence of decelerations, and total time of decelerations demonstrated good or moderate interobserver agreement, kappa ranged 0.47–0.80, indicating fairly high consistency in estimating these parameters. The kappa coefficients for these features ranged from moderate to very good levels. The assessment of accelerations exhibited only weak to moderate concordance (kappa: 0.29–0.47). Evaluation of the deceleration type yielded the lowest agreement. The overall categorization of CTGs into categories exhibited only poor to moderate interobserver concordance (Fleiss kappa: 0.19–0.44).

**CONCLUSIONS:**

CTG parameters – baseline rate, variability, presence/absence of decelerations, and total width of decelerations in a 30-minute CTG interval – are features that can be interpreted with a high degree of objectivity and agreement with appropriate training, even without clinical experience. Since the categorization of CTGs into separate categories (normal, suspicious, and pathological) has a poor to moderate level of agreement, it indicates a need for discussion on whether it is worth continuing to rely on such CTG categorical stratification or base CTG judgements on more objective and high agreement parameters.

## INTRODUCTION

Since its initial implementation in 1968, electronic fetal monitoring via cardiotocogram (CTG) has aimed to facilitate early detection of fetal hypoxia, thereby permitting timely interventions to prevent adverse neonatal outcomes. Despite its widespread adoption, the predictive value of CTG features remains limited, with studies indicating a high negative predictive value but a low positive predictive value for fetal hypoxic injury^1^. Moreover, the subjectivity inherent in interpreting CTG patterns leads to significant interobserver variability, which poses challenges to consistent clinical management. Previous guidelines, notably those issued by The International Federation of Gynecology and Obstetrics (FIGO), have undergone multiple revisions, with the most recent being in 2015, aiming to standardize interpretation criteria based on fetal physiological parameters^2^. Despite these efforts, variability persists, especially regarding deceleration interpretation, accelerations, and overall categorization, which may contribute to diagnostic inaccuracies and inconsistent clinical responses^3^. The overall assessment of a CTG as normal, suspicious, or pathological, and making the right decision at birth, remains one of the greatest challenges. Increasingly, it is argued that it would be appropriate to define individual CTG parameters that have both good interobserver agreement and the best prognostic value in assessing the fetal condition. One example of such a parameter could be the total width of decelerations in a 30-minute CTG interval, given that it is the duration of deceleration that has a very good prognostic value in predicting neonatal acidosis^4,5^. The proposed parameter is likely to be easy to estimate and the possibility of misinterpretation is low. If this objectively easy-to-measure parameter also has a good negative and positive predictive value in assessing the fetal status at the time of investigation, it could be an ideal CTG parameter.

It has been reported that in the presence of meconium-stained amniotic fluid, even after emergency cesarean section, a high proportion of newborns are born in a serious condition^6,7^. This shows that the current interpretation of the CTG makes it difficult to predict the actual fetal condition and to make a timely decision. For this purpose, we wished to assess if the evaluation of individual CTG parameters could more accurately predict fetal condition. The aim of this study was to assess interobserver agreement among investigators in assessing fetal status during labor using selected CTG parameters, according to the FIGO 2015 CTG assessment guidelines^2^. We evaluated whether the assessment of individual CTG parameters is more objective and has better inter-investigator agreement than assigning the entire CTG to separate categories (normal, questionable, and pathological). The second hypothesis is that the clinical experience of investigators positively affects the interpretation of CTG parameters. Addressing these hypotheses may serve as the starting point for a future study on the assessment of fetal status in meconium-contaminated amniotic fluid.

## METHODS

This multicenter interobserver agreement study involved two leading Lithuanian maternity institutions, with ethical approval obtained from the local ethics committee. The study took place in the Hospital of Lithuanian University of Health Science Kauno Klinikos (tertiary maternity unit) and Klaipeda University Hospital (maternity unit, together with a midwife-led unit), both having about 3000 deliveries per year. Study period was April 2024. Delivery records from term singleton pregnancies presenting with meconium-stained amniotic fluid and without major fetal anomalies or complicating factors were included. A total of 84 CTGs (50 and 34 from different hospitals), each with a minimum duration of 120 minutes during the active labor phase, were randomly selected for detailed analysis. These recordings were subdivided into four equal segments of 30 minutes each for comparative evaluation. Only recordings of adequate quality with no artifacts were considered. CTG with poor quality tracings, mostly related to the pushing stage of birth and the different pushing positions used, were not considered.

Six clinicians, each blinded to clinical outcomes, independently assessed the CTGs on standardized parameters outlined by the FIGO 2015 guidelines^2^ – specifically, baseline fetal heart rate, variability (categorized as normal, reduced, or absent), presence and nature of decelerations, their morphology and type, total deceleration duration, and bradycardia. The observers, comprising both highly experienced practitioners (>10 years, three doctors, obstetricians, and gynecologists, including those from both hospitals) and less experienced trainees (three young doctors, residents with <3 years’ experience from the teaching university hospital), received prior standardized training on the guidelines. The evaluators had no time limit for the assessments, and in each hospital they analyzed the same cardiotocographic traces.

The manuscript was prepared in accordance with the GRASS to ensure transparent and comprehensive reporting. Data analysis involved calculating Cohen’s and Fleiss’ kappa statistics to determine interobserver agreement, complemented by ICC for interval data such as deceleration duration. Kappa values were interpreted according to established categories (weak <0.20; poor 0.21–0.40; moderate 0.41–0.60; substantial 0.61–0.80; almost perfect ≥0.80). PA was interpreted using conventional thresholds, with >80% agreement considered acceptable.

### Data management

All collected data were stored, processed, and managed in accordance with institutional data protection policies. We confirm that the security and confidentiality measures applied fully comply with these institutional standards.

### Ethical approval

Ethical approval was obtained from Kaunas Regional Biomedical Research Ethics Committee (number BE-2-61, approved on 5 May 2024). All participants on admission filled in and signed the hospital’s patient consent form for the use of their data for scientific purposes. Written informed consent from the patients was not required to participate in the present study, in accordance with the national legislation and the institutional requirements. The data security and confidentiality measures comply with institutional policies.

## RESULTS

The data are summarized in Table 1. A total of 663 variables were analyzed to determine interobserver reliability. The findings demonstrated that most CTG parameters – specifically, variability, bradycardia, presence or absence of decelerations, reduced variability accompanied by tachycardia, and the total deceleration duration – exhibited good to moderate levels of agreement. These parameters yielded kappa coefficients within the range of moderate to very good concordance (kappa: 0.67–1). Conversely, assessments pertaining to accelerations showed only moderate or poor agreement (kappa: 0.29–0.47). The evaluation of deceleration type and morphology (shape) showed the lowest interobserver concordance, with kappa values between 0.12 and 0.23, indicative of poor or weak agreement.

**Table 1:** The results of interobserver agreement on different CTG parameters

*Variables*	*T1–T2 observers*			*T3–T6 observers*		
	*n*	*Kappa coefficient for individual variables (95% CI)*	*Fleis kappa coefficient (95% CI)*	*n*	*Kappa coefficient for individual variables (95% CI)*	*Fleis kappa coefficient (95% CI)*
**Variability**						
Normal	50	0.67 (0.39–0.94)	0.67 (0.39–0.95)	34	0.62 (0.48–0.76)	0.62 (0.48–0.76)
Reduced		0.67 (0.39–0.95)			0.61 (0.48–0.75)	
**Accelerations**						
Present	50	0.47 (0.33–0.61)	0.47 (0.33–0.61)	34	0.46 (0.33–0.60)	0.47 (0.33–0.61)
Absent		0.29 (0.02–0.57)			0.47 (0.33–0.61)	
**Decelerations**						
Present	50	0.69 (0.42–0.97)	0.69 (0.42–0.97)	34	0.52 (0.38–0.66)	0.53 (0.39–0.67)
Absent		0.69 (0.42–0.97)			0.53 (0.39–0.67)	
**Deceleration type**						
Variable	29	0.19 (-0.17–0.56)	0.23 (-0.07–0.54)	34	0.13 (0.05–0.32)	0.13 (0.02–0.27)
Early		0.37 (0.01–0.073)			0.13 (0.05–0.32)	
Late		-0.06 (-0.42–0.31)			0.12 (0.07–0.30)	
**Shape of decelerations**						
V shaped	29	0.13 (0.02–0.42)	0.12 (0.07–0.37)	19	0.22 (0.04–0.41)	0.22 (0.08–0.39)
U shaped		0.03 (-0.33–0.39)			0.32 (0.14–0.51)	
W shaped		-0.07 (-0.43–0.29)			0.01 (-0.18–0.19)	
Bradycardia	50	1 (N/A)		34	1 (N/A)	
**Tachycardia and reduced variability**						
Present	50	0.78 (0.67–1.00)	0.79 (0.67–1.00)	34	0.58 (0.44–0.72)	0.57 (0.43–0.71)
Absent		0.78 (0.67–1.00)			0.57 (0.43–0.71)	
**CTG evaluation**						
Normal	50	0.48 (0.20–0.76)	0.44 (0.18–0.70)	34	0.22 (0.08–0.36)	0.19 (0.06–0.32)
Suspicious		0.42 (0.14–0.69)			0.17 (0.04–0.31)	
Pathological		-0.01 (-0.29–0.27)			-0.02 (0.15–0.12)	
**Total width of decelerations in a 30-min CTG interval**						
	***n***	***ICC (95% CI)***		***n***	***ICC (95% CI)***	
	29	0.48 (0.14–0.72)		19	0.80 (0.64–0.90)	

ICC: interclass correlation coefficient.

The overall classification of CTGs into categories– normal, suspicious, or pathological – demonstrated limited reproducibility; specifically, Cohen’s kappa for the T1–T2 group was 0.44, reflecting moderate agreement, whereas the other evaluator group (T3–T6) exhibited only minimal agreement (kappa: 0.19). When stratified according to the Lithuanian national guidelines, the assessment of CTG categories (normal, suspicious, or pathological) resulted in kappa values of approximately 0.48, 0.42, and 0, respectively, for T1–T2, and only weak or poor agreement ranging from 0.22 to 0 among T3–T6.

## DISCUSSION

Application of the 2015 FIGO CTG scoring criteria^2^ yielded moderate to high interobserver agreement for parameters such as baseline fetal heart rate, variability, presence or absence of decelerations, and total deceleration duration, corroborating previous findings by Rei et al.^8^. These features are comparatively straightforward to interpret objectively following standardized training, thereby minimizing subjective variability. In contrast, assessments of accelerations demonstrated lower interobserver concordance (kappa: 0.29–0.47) in our cohort compared to prior studies (kappa: approximately 0.72)^9,10^, potentially attributable to recent literature highlighting interpretative ambiguities – for instance, the differentiation of true versus pseudo-accelerations – confusing clinical assessment^8^. The work by Al Fahdi et al.^10^ has underscored that misinterpretation of accelerations may impact reliability, which could explain the lower kappa values observed. Recent evidence^11^ further supports this, reporting acceleratory agreement as low as 0.09 (poor).

Furthermore, the agreement concerning the classification of deceleration types (early, late, variable) was similarly low across studies, with coefficients ranging from 0.19 to 0.38^8,9,12^. Our findings align with this trend, yielding kappa values of only 0.12–0.23, indicating substantial interobserver variability.

The overall CTG scoring – differentiating between normal, suspicious, and pathological – demonstrated moderate agreement between experienced observers (T1–T2; kappa: 0.44), consistent with prior research, such as that of Rei et al.^8^ who reported kappa=0.60. However, less experienced examiners (T3–T6) only achieved poor to weak concordance (kappa: 0.19). This discrepancy underscores the necessity of continuous education, ongoing training initiatives, and long-term mentorship to enhance the consistency and accuracy of CTG interpretation.

Notably, the classification of CTGs into categories (normal, suspicious, and pathological) was particularly unreliable. Kappa values between T1 and T2 ranged from 0 to 0.48 for this parameter: normal CTG 0.48 (moderate agreement), suspicious CTG 0.42 (moderate agreement) and pathological CTG 0 (poor agreement). The results were significantly worse for T3, T4, T5 and T6, with poor to weak agreement in the assignment of CTGs to individual categories, ranging from 0 to 0.22 (normal 0.22, suspicious 0.17 and pathological 0). The results are also surprising in that they contradict the notion that normal and pathological CTGs are the easiest to detect, while the category of suspicious CTG is more difficult to identify. International studies indicate the best interobserver agreement in the normal CTG group (kappa: 0.67–0.71) and in the pathological CTG group (kappa: 0.57), while suspicious CTG had a lower agreement (0.36–0.52)^8,13^. In our study, all observers had trouble reaching a consensus on the pathological CTG (kappa: 0). Given that the identification of a pathological CTG is critical for urgent decision-making, these findings raise concerns regarding the current reliability of categorical assessments.

An innovative aspect of this study was the emphasis on the objective quantification of deceleration duration. The calculated kappa values, ranging from 0.48 to 0.80, demonstrate that estimation of total deceleration duration is one of the most reliably interpreted parameters of CTG with minimal influence from clinician experience and enhances the case for its routine and objective measurement in clinical practice. Cahill et al.^4^ have shown very convincingly in their study that the duration of deceleration has the best prognostic value in predicting neonatal acidemia compared with other CTG parameters^4^. Although decelerations are one of the most readily identifiable CTG features with the best prognostic value, it should be noted that high agreement is achieved by assessing whether decelerations are present and how much time the fetus spends in deceleration, but not by assessing the types of decelerations. The debate about the value of different decelerations arises from the work of Xodo and Londero^14^ who refuted the correlation of distinct types of decelerations with distinct physiological mechanisms for their occurrence. The mentioned authors argue that there is no better prognosis for so-called ‘physiological’ decelerations, and that all decelerations are ‘bad’ and have a negative impact on the fetal condition. The fetal deterioration and the development of acidemia are not determined by the type of deceleration (variable, early, late), but by its duration and the residual time of the basal rate.

### Strengths and limitations

A strength of the study is the participation of two maternity hospitals, which allows us to ensure that the results are not a reflection of the clinical practices of a single facility. Standardized training for all observers was provided prior to the study to ensure that the CTGs would be evaluated in the same manner according to the selected parameters. The analysis of the study also included CTGs recorded in the last 30 min before delivery, which is the most challenging CTG to assess, and may partly explain the results obtained in this study, which showed that the assessment of even basic parameters such as baseline rate or variability does not achieve very good agreement and remains a challenge for clinicians working in labor wards.

A limitation of this study is its retrospective design; the observers were able to assess the CTGs without limiting the assessment time and in a quiet environment, using the CTG assessment guidelines. Such an assessment is quite dissimilar to day-to-day clinical work, where CTGs need to be assessed on the labor ward, at times during the night and in a rush, and a prospective study would have allowed for potentially even greater differences in agreement to be observed.

## CONCLUSIONS

The overall evaluative reliability of CTG interpretation demonstrates only moderate to low interobserver concordance. Key CTG parameters – including baseline fetal heart rate, variability, presence or absence of decelerations, and the duration of decelerations – are features that can be reliably quantified and interpreted with high reproducibility following standardized training, independent of clinical experience.

Given the limited agreement observed in classifying CTGs into categorical designations (i.e. normal, suspicious, or pathological), it would be indicated to discuss whether it is worth continuing to rely on such CTG categorical stratification. Instead, clinical decision-making should primarily be guided by more objective, reproducible features – such as baseline rate, variability, bradycardia, presence or absence of decelerations, and the cumulative deceleration area within a defined interval (e.g. 30 min) –which demonstrate higher interobserver reliability and prognostic validity.

## Data Availability

The data supporting this research are available from the authors on reasonable request.
